# BMI and the Food Retail Environment in Melbourne, Australia: Associations and Temporal Trends

**DOI:** 10.3390/nu15214503

**Published:** 2023-10-24

**Authors:** Cindy Needham, Claudia Strugnell, Steven Allender, Laura Alston, Liliana Orellana

**Affiliations:** 1 Global Centre for Preventive Health and Nutrition, Institute for Health Transformation, Deakin University, Geelong, VIC 3220, Australia; claudia.strugnell@deakin.edu.au (C.S.); steven.allender@deakin.edu.au (S.A.); 2 Institute for Physical Activity and Nutrition (IPAN), Deakin University, Geelong, VIC 3220, Australia; 3 Colac Area Health, Colac, VIC 3250, Australia; laura.alston@deakin.edu.au; 4 Faculty of Health, Deakin Rural Health, Deakin University, Warrnambool, VIC 3280, Australia; 5 Faculty of Health, Biostatistics Unit, Deakin University, Geelong, VIC 3220, Australia; l.orellana@deakin.edu.au

**Keywords:** food environment, public health nutrition, BMI, obesity, food retail

## Abstract

Research into the link between food environments and health is scarce. Research in this field has progressed, and new comprehensive methods (i.e., incorporating all food retail outlets) for classifying food retail environments have been developed and are yet to be examined alongside measures of obesity. In this study, we examine the association and temporal trends between the food environment and BMI of a repeated cross-sectional sample of the adult population between 2008 and 2016. Methods: Food retail data for 264 postal areas of Greater Melbourne was collected for the years 2008, 2012, 2014, and 2016, and a container-based approach was used to estimate accessibility to supermarkets, healthy and unhealthy outlets. Data on BMI for postal areas was obtained from the Victorian Population Health Survey (n = 47,245). We estimated the association between the food environment and BMI using linear mixed models. Results indicated that BMI increased as accessibility to healthy outlets decreased by up to −0.69 kg/m^2^ (95%CI: −0.95, −0.44). BMI was lower with high and moderate access to supermarkets compared to low access by −0.33 kg/m^2^ (−0.63, −0.04) and −0.32 kg/m^2^ (−0.56, −0.07), and with high access to unhealthy outlets compared to low access (−0.38 kg/m^2^: −0.64, −0.12) and moderate access (−0.54 kg/m^2^: −0.78, −0.30). Conclusion: Our results show that increasing access and availability to a diverse range of food outlets, particularly healthy food outlets, should be an important consideration for efforts to support good health. This research provides evidence that Australia needs to follow suit with other countries that have adopted policies giving local governments the power to encourage healthier food environments.

## 1. Introduction

The World Health Organization’s (WHO) Global Action Plan for the Prevention and Control of Noncommunicable Diseases 2023–2030 has a target of halting the rise in the prevalence of people with overweight and obesity by 2030 [1]. To achieve this, the plan highlights the importance of strengthening the capacity of populations to make healthier choices through providing supportive environments [1]. The food environment has the potential to support healthier choices through different elements, including food access and availability, promotion, labelling, and price [2,3,4,5]. The food retail environment (FRE), which refers to the accessibility and availability of food retail outlets (i.e., where and what foods can be obtained, respectively) within neighbourhoods, is a key focus for obesity prevention efforts as they have the potential for sustained community-level impact [3,4,5].

The inclusion of policies that support promoting a healthier FRE is one approach that would assist decision makers in planning healthier urban environments that support healthy choices [2]. Guided by recommendations from the National Institute for Health and Care Excellence [6,7], Public Health England has included within the planning system powers for local authorities to restrict planning permission for takeaways and other food retail outlets in specific areas (i.e., near schools or in areas of deprivation and high obesity prevalence) [8]. Using these planning powers, local authorities in North England have been successful in decreasing the density of unhealthy food outlets (i.e., hot food takeaways) [9]. In Australia, little success has been achieved at the policy level to regulate the healthiness of FRE. Commercial pressures and the absence of strong context-specific evidence to support the need for policy are two barriers to FRE policy development [5].

Lack of investment in nutrition research in Australia may also explain some of the evidence gaps in the link between FRE, diet, and health [10]. A systematic review of studies examining the FRE in Australia identified only 13 of 60 studies (published between 2008 and 2019) that examined the association between the FRE and the prevalence of obesity [11,12,13]. The majority of studies take a narrow view, examining the FRE cross-sectionally and using only measures of supermarkets or fast-food outlets to represent the FRE [11]. Research in this field has progressed, and new comprehensive methods (i.e., incorporating all food retail outlets) for classifying FRE have been developed and are yet to be examined alongside measures of obesity [14].

The review also brought to the forefront the lack of longitudinal studies examining the FRE alongside health measures [11]. Despite evidence of rapid change in the FRE over time in Australia [15], of five Australian longitudinal studies examining the association between healthy weight and the FRE, only one used time-sensitive measures of the FRE, examining only the top 10 fast-food chains in association with BMI [16]. The remaining four used a single measure of the FRE alongside longitudinal obesity-related measures [17,18,19,20]. Studies that use measures of both obesity and the FRE over time have the potential to add value to the research by demonstrating whether relationships remain consistent over time [11,21].

Therefore, this study sought to:

1. Examine the association between the FRE at the postal code level and the BMI of a repeated cross-sectional sample of the adult population residing in those postal codes at four time points (2008, 2012, 2014, and 2016);
2. Examine temporal trends at four time points over eight years in BMI across measures of the FRE.

## 2. Materials and Methods

### 2.1. Design

This is a secondary analysis of data from a repeated cross-sectional sample of the Victorian population and an audit of food outlets, both collected in 2008, 2012, 2014, and 2016. BMI data were obtained from the Victorian Population Health Survey (VPHS) [22]. The Victorian Department of Health’s Human Research Ethics Committee granted ethics approval for the VPHS [23]. The lead author’s institution granted an ethics exemption for the present study.

### 2.2. Study Region

The setting of the study is Greater Melbourne (Melbourne), Australia. Melbourne is the capital city of Victoria and has experienced rapid population and urban growth at a rate greater than any other Australian city [24,25]. Between 2008 and 2016, Greater Melbourne’s population increased from 3.9 million to 4.9 million [24,25]. In 2016, 49% of the population was male and 51% was female [25]. Children (aged 0–14 years) made up 18.3% of the population, and people aged over 65 years made up 14%, with the median age of people being 36 years [25]. The study area included 264 postcodes.

### 2.3. Victorian Population Health Survey

The VPHS is an annual cross-sectional telephone interview survey [23]. The VPHS collects self-reported data on demographics, health status, behaviours, and risk factors [26]. We used the 2008, 2011–2012, 2014, and 2017 expanded surveys, each including approximately 34,000 participants across Victoria [27].

### 2.4. Participants

The VPHS collects unidentified individual-level data on the health status of a random sample of adults aged 18 years or more [28] using computer-assisted telephone interviews (CATI) with sampling via random digit dialling [27]. The sample is stratified by local government area (LGA), with a target of 426 interviews per LGA [29]. All data are self-reported and stored within the CATI system [28]. The sampling frame has changed over time to accommodate the increasing proportion of the population that does not have a landline telephone (i.e., mobile-phone-only households) [23]. In 2008, 2012, and 2014, the sampling frame only included landline numbers, whereas from 2015 onwards, a dual-frame sampling design was utilised with randomly generated samples of both landline and mobile phone numbers. Using this design, a larger proportion of persons likely to be mobile-only users can be included in the survey [23].

Participants who lived in one of the 264 (excluding the CBD) residential postcodes located in Melbourne in 2008, 2012, 2014, and 2017 were included in the study sample (n = 47,245). The central business district (postcode 3000) was excluded as food outlet data for this area was not collected.

### 2.5. Exposure Variables—Food Retail Environment

#### 2.5.1. Data Collection and Definition of Measures

Food outlet data (food outlet name, type, and address) were extracted retrospectively from hard copy business directories (Yellow and White Pages) for the years 2008, 2012, 2014, and 2016 to align with the available expanded population health data from the VPHS. A copy of the Yellow Pages was not available for the year 2017. As such, food outlet data from 2016 was used to represent food retail environment exposure variables for 2017 VPHS participants (referred to as 2016 data from hereon). Melbourne’s central business district has a very large number of food outlets, predominantly servicing workers and visitors, and therefore was not collected [30]. The methods used to classify food outlets have been described elsewhere [15]. In short, we adapted an Australian [31] food outlet classification tool to classify outlets into one of 17 types [15] and allocated a score representing healthiness using a 21-point scoring system ranging between −10 (least healthy) and +10 (most healthy) (Supplementary Table S1) [15,31]. Outlets were classified as (1) healthy (healthiness score: +5 to +10), including supermarkets, fruiterers and greengrocers, butchers, fish and poultry shops, and salad/sandwich/sushi bars; (2) less healthy (healthiness score: −4 to +4), including cafes and restaurants (independent and franchise), bakers, and delis; and (3) unhealthy (healthiness score: −10 to −5), including fast-food outlets, independent takeaways, pubs, general stores, and specialty extra.

#### 2.5.2. Geographical Area Level Definition

The addresses of the food outlets were mapped against the 2016 postal area (POA) boundaries of the Australian Bureau of Statistics (ABS) [32]. Food outlet exposure measures (outlets per km^2^ and relative healthy food availability) were then calculated for the 264 postal areas that were entirely within Melbourne. In this study, the POA is considered the ‘activity space’ in which the majority of food retail exposure and purchasing occurs [33].

#### 2.5.3. Food Retail Environment Measures of Accessibility and Availability

We used the most common approach, referred to as the ‘container-based method’ or ‘statistical index approach’, to estimate food outlet accessibility [34,35]. In this method, the number of food retail outlets within a geographic unit is summarised (e.g., the number and total area of density within a specific geographic unit) [35]. The approach was selected due to its suitability for comparison across regions and over time [34]. Using this method, the FRE of each POA was characterised using four food retail accessibility measures (FRAMs) and a measure of relative healthy food availability (RHFA) [3,18]. FRAMS represent accessibility defined as the number of food outlets within a POA (the container) per km^2^ for: (1) supermarkets; (2) healthy; (3) less healthy; and (4) unhealthy outlets [14]. Supermarkets were considered independently given that they account for the bulk of food retail purchases (68% in 2019) in Australia [36]. Each FRAM measure was then categorised into three levels (Table 1). Less healthy outlets and RHFA were used to inform typology (outlined below), but their association with BMI was not assessed, as less healthy outlets are not considered highly influential on health in the Australian literature [11], and RHFA alone does not reflect the potential accessibility of outlets [37]. RHFA was calculated as the number of food outlets classified as supermarkets, and fruiterers and greengrocers (as a proxy for healthy food outlets) relative to the number of supermarkets, greengrocers plus fast-food franchise and independent takeaway outlets (considered a proxy for unhealthy outlets) [3,18]. The RHFA was categorised into four levels [18] (Table 1). The FRE of POAs was also classified into nine typologies based on the FRAM measures and RHFA, following the approach described by Needham et al. [14]. For POAs with zero food outlets, the FRAMS for the Statistical Area 2 (i.e., suburbs and residential districts) in which the POA was located was used to calculate ‘Typology’ [38,39].

### 2.6. Outcome Measure and Potential Confounders

The outcome measure was BMI (weight (kg)/height^2^ (m^2^)) determined from VPHS participant self-reported height and weight. Several potential confounders (age, gender, education, annual household income, and employment status) of the relationship between FRE and BMI were considered based on previous literature [11] (Table 2). The variable ‘length of time the participant had lived in the current neighbourhood/area/council/local government area (LGA)’ (categorical) was used as a proxy for duration of exposure to the local FRE.

### 2.7. Statistical Analysis

Participants were assigned the FRE exposure measures calculated based on their residential POA in the corresponding calendar year. Linear mixed models were fitted to estimate the mean BMI of participants across exposure levels and study years. All models included the year (categorical), the exposure measure (categorical), the interaction exposure by year, and the potential confounders as fixed effects. The models also included POA as a random effect. Where the interaction exposure by time was non-significant, Sidak-adjusted pairwise comparisons were reported between levels of each factor (exposure and year). Stata version 15.0 was used for all statistical analyses.

## 3. Results

### 3.1. Food Environment Characteristics

Supermarket and healthy outlet accessibility was low for most POAs and decreased over time. Almost half of the POAs had high access to unhealthy and less healthy outlets in 2008, increasing across the study period. Of the nine potential typologies, seven were identified across POAs. The two more frequent typologies were High access—Low % healthy and Low Access—Low % healthy. Across study years, forty-three POAs had zero food outlets in at least one of the years (2008: N = 38, 2012: N = 37, 2014: N = 31, 2016: N = 31). Twenty-one SA2′s housed the forty-three POAs with zero food retail.

Supplementary Table S2 presents the proportion of POAs that are within each food retail classification. Supplementary Table S3 presents the descriptive statistics for each FRE typology. Supplementary Table S4 presents the proportion of participants within each FRE measure classification.

### 3.2. Sample Characteristics

The four waves of the VPHS included 52,498 participants residing in Melbourne. Cases were excluded due to missing data for height (n = 1585), weight (n = 3070), education (n = 368), employment status (n = 134), or length lived in the local area (n = 44). A further 52 participants were excluded due to implausible height or weight measures, or where BMI was extreme (BMI ≥ 70). The final analysis sample included 47,245 participants. Characteristics of the sample are provided in Table 2.

### 3.3. Relationship between BMI and Food Retail Environment Measures

**Figure 1:**
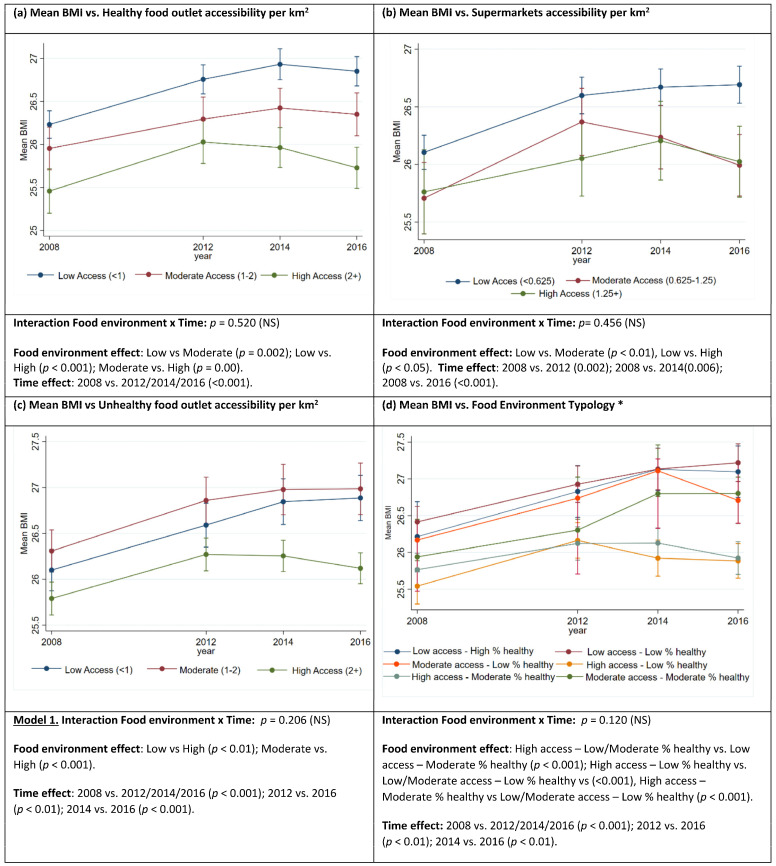
(**a**–**d**). Mean body mass index over the period 2008–2016 across postal areas grouped by food environment exposure variables. Mean BMI estimates and 95% confidence intervals were obtained under linear mixed models including postal area as a random effect, and the food environment measure (healthy, supermarkets, unhealthy food outlets, and combined measure of all outlets ‘Typology’), year, interaction food environment measure by year adjusted by age, gender, education, employment status, household income, and length of time lived in the local area. Only pairwise comparisons where *p* ≤ 0.05 are reported. * Food environment typology reflects postcodes grouped by similarities across relative healthy food availability (RHFA); and food retail accessibility measures to supermarkets, healthy, less healthy, and unhealthy food outlets per km^2^. RHFA: represents the proportion (%) of the food environment that is composed of supermarkets and greengrocers (as a proxy for healthy food) from the sum of supermarkets, greengrocers, independent takeaway, and fast-food franchise outlets within each postal area boundary. Body mass index (BMI) = weight (kg)/height^2^ (m^2^)).

The mean BMI profile across the study period was the same for different levels of FRE measures (Figure 1). Sidak-adjusted pairwise comparisons (*p* < 0.05) between exposure levels and between years are reported in Figure 1a–d and Supplementary Tables S5 and S6. There was a significant difference in mean BMI across levels of accessibility to healthy food outlets, with BMI progressively increasing as accessibility to healthy outlets decreased (Figure 1a). BMI was lower in areas with high access to healthy food outlets compared to low access (−0.68 kg/m^2^, 95%CI: −0.94, −0.43) and moderate access (−0.34 kg/m^2^; −0.60, −0.80); BMI was also lower in areas with moderate access compared to low access (−0.34 kg/m^2^; −0.57, −0.12). BMI was lower for people living in POAs with high and moderate access to supermarkets when compared to low access by −0.32 kg/m^2^ (−0.64, −0.12) and 0.33 kg/m^2^ (−0.63, −0.04) (Figure 1b). BMI was lower in POAs with high access to unhealthy outlets compared to low access (−0.38 kg/m^2^; −0.64, −0.12) and moderate access (−0.54 kg/m^2^; −0.78, −0.30) (Figure 1c).

The mean BMI was significantly different across FRE typologies (Figure 1.d). BMI was lower in POAs classified High access–Moderate % healthy (−0.73 kg/m^2^; −1.08, −0.39) and High access–Low % healthy (−0.84 kg/m^2^; −1.2, −0.47) when compared to Low access–Low % healthy. Mean BMI was also lower in High access–Low % healthy (−0.73 kg/m^2^; −1.2, −0.27) and Moderate % healthy (−0.63 kg/m^2^; −1.08, −0.19) when compared to Low access–Moderate % healthy. This was also the case when High access–Moderate % healthy (−0.57 kg/m^2^; −0.94, −0.2) and High access–High % healthy (−0.67 kg/m^2^; −1.05, −0.29) were compared to Moderate access—Low % healthy.

### 3.4. Temporal Trends in BMI

The mean BMI of participants increased across all measures of the food retail environment over the study period by as much as 0.73 kg/m^2^ (0.51, 0.95) (Figure 1a–d, Supplementary Tables S5 and S6).

## 4. Discussion

This study provides the first evidence of a temporal relationship between self-reported BMI and the FRE. A lower BMI was consistently associated with higher access to supermarkets, healthy and unhealthy outlets over the 8-year study period. Using the combined measure ‘typology’ demonstrated that over half of the postal areas had less than 25% healthy outlets and that access tended to be similar (i.e., where access was high, it was high for all outlets). Findings suggest that having more than 50% healthy outlets in low-access POAs and more than 25% healthy outlets in moderate-access POAs may play some role in facilitating the healthier weight of residents.

Our findings are consistent with earlier studies reporting a relationship between a lower adult BMI and access to healthy outlets in Sydney, Australia [18]. In this study, a higher BMI was associated with FRE, where unhealthy outlets made up more than 25% of all outlets (healthy and unhealthy outlets) within 1.6 and 3.2 km from home [18]. For children in Perth (Australia), decreasing BMI with increasing access to healthy outlets was also reported, with every additional healthy outlet within an 800 m and 3 km buffer from home corresponding with a reduction in risk of being overweight or obese by 19% and 2%, respectively [40]. Our findings are also supported by earlier evidence that suggests good access to supermarkets is associated with a lower BMI for adults, significant within 2 km from home [41].

An inverse relationship between access to unhealthy outlets and BMI is also reflected in earlier studies examining dietary behaviours among women, with results indicating women reporting that they never consume fast food had a higher density and variety of fast food near home in Melbourne (measured as proximity and density within 3 km) [42]. In this 2010 study, access to both fast-food and healthy outlets was highly correlated, and increased fruit and vegetable consumption was also associated with increased access to supermarkets and greengrocers [42]. Another earlier Australian study reported an inverse relationship between the density of fast food near home (2 km) and healthy weight (BMI) in both children and their male parent [43]. The finding that greater access to unhealthy food is associated with a lower BMI should be interpreted with caution. Not least because, evidence from other studies reports a positive association between unhealthy food access, consumption, and unhealthy weight [19,44,45].

An explanatory pathway as to why there is a relationship with a lower BMI where healthy and unhealthy food outlets are more plentiful is that residents have fewer barriers to accessing healthier food and a lower time–cost associated with purchasing food, resulting in more time available for food preparation and a decreased reliance on unhealthy convenience foods [46,47]. This is supported by qualitative studies that suggest those with poor access to food retail develop adaptive shopping behaviours, purchasing food in bulk and selecting products with a longer shelf life (i.e., fewer fresh items, more non-perishable/frozen items), with car ownership and food insecurity further found to accentuate these adaptive behaviours [47,48].

These findings highlight a need to further unpack the drivers of consumption patterns and healthy weight from within the complex FRE using measures, like that used in our study, that take into consideration the full breadth of food outlet types to examine the potential confounding factors at play within the FRE.

### 4.1. Strengths

This is the first repeated cross-sectional study examining the relationship between BMI and comprehensive longitudinal measures of the FRE alongside BMI. Our findings are strengthened by removing potentially confounding variables, adjusting for socioeconomic differentials, and using a large, comprehensive, and representative sample. Despite some reported discrepancies, correlations between self-reported and measured height and weight were strong in adults (aged ≥ 45) [49].

### 4.2. Limitations

The duel-frame sampling design introduced in 2015 may have caused some variability in sample selection over the study period [28]. While the VPHS aims to capture a representative sample of the population from each LGA, the differential self-selection of those willing to participate in the survey cannot be ruled out. As a consequence, the samples may not have been representative of the broader population, which may limit the generalizability of the findings. We were also unable to account for other dynamic factors that could influence BMI trends, such as population shifts in dietary preferences, economic conditions, or public health campaigns that may have occurred over the study period. Self-reported height is often overreported, and self-reported weight is often underreported, leading to an overall, but likely uniform, underestimation of BMI [49]. De-identification of the VPHS data meant that the geocoded location of each participant household was not available, and as such, only broad measures of accessibility to food outlets (count per km^2^) within each POA could be calculated. Our measure of access assumes food resources and people are evenly spread across POAs; it is likely that food resources cluster around residential areas within POAs and not all residents have the same accessibility. The use of POA in this sample and in geographical research broadly can be subject to a modifiable areal unit problem [50], where the size and shape of different areas can change the picture that the data conveys [50]. Finally, this study does not take into account FRE outside of the residential POA, such as at work, school, or in transit, nor does it include other potential confounders such as physical activity levels and available physical activity infrastructure or other neighbourhood characteristics, or participants’ health conditions, which may also influence BMI [51]. While the findings from this study do not imply causation, the comprehensive measures used in the study at the residential POA level provide for a more accurate representation of FRE exposure than reported in earlier studies, and the consistency of results at multiple time points suggests there may be a relationship between population-level estimations of the FRE and BMI.

### 4.3. Implications for Population Health Policy and Research

This study is of international significance to countries experiencing rapid population growth and urban expansion, providing insight into the higher mean BMI of residents in areas with lower accessibility to a diverse range of food outlets. Future research on longitudinal health and anthropometric (i.e., weight and height) data from a large-scale sample of the population, alongside comprehensive repeated measures of the FRE such as in this study, would provide stronger evidence of the link between the FRE and health. However, given the relationship between healthier BMI and high access to healthier food retail outlets, the findings provide a rationale for policies that aim towards the development of compact cities to support health and encourage increasing accessibility to healthier (healthy and less healthy) food outlets over unhealthy outlets. For policy and planning to gain traction, ‘health’ needs to be included as a consideration within planning legislation, a central issue raised in an earlier study (December 2016 and August 2017), which undertook interviews with government, non-government, and private stakeholders in Melbourne [52]. Evidence, along with international progress, supports incorporating standards into urban planning guidelines that seek to encourage access to healthy outlets within 1 km of most homes [53]. Where this is not economically viable, facilitating transport opportunities to existing healthy food resources and investing in diverse healthy food retail opportunities (e.g., healthy food delivery services or greengrocer pop-up stores) for residents, particularly those without vehicle access, needs to be explored [48]. Results also suggest that having unhealthy outlets make up no more than 25% of outlets in moderate-access areas and no more than 50% in low-access areas may mitigate the effects of lower accessibility to food retail overall. Future research would benefit from understanding how individuals interact with their FRE, their decision making around the use of one food retailer over others, their purchasing patterns, and how this translates to actual dietary intake [5,40]. Finally, reliable longitudinal FRE data (i.e., routine monitoring) examining all food outlets within neighbourhoods is needed; this will provide for further linkage opportunities with population health data, which will support future research and decision making [11].

## 5. Conclusions

A consistent relationship exists between self-reported BMI and healthier FRE characteristics. Our results show that increasing access and availability to a diverse range of food outlets, particularly healthy food outlets, should be an important consideration for efforts to support the evolution of healthy weight environments. This research provides evidence that Australia needs to follow suit with other countries that have adopted policies that give local governments the power to encourage healthier FRE. To further understand what the moderators and mediators of healthy weight are from within the FRE, further exploration into the lived experience of the FRE across geographic and socioeconomic differentials over time is required.

## Figures and Tables

**Table 1:** Classification of food retail environment accessibility measures.

Measures			Classification
**Relative healthy food** **availability (RHFA)**	**Proportion healthy food** **resources**		**Availability**
	No food retail * ≤25%		Low
	>25 to-≤50%		Moderate
	>50%		High
**Food retail accessibility measures (FRAMs)**	**Count per km^2^**		**Access**
Healthy, less healthy, unhealthy	<1		Low
	≥1 to <2		Moderate
	≥2		High
Supermarkets	<0.625		Low
	0.625 to <1.25		Moderate
	≥1.25		High
Food environment typology **	FRAMs ***	**RHFA**	**Typology**
	Low	≤25%	Low access—Low % healthy
	Low	>25% to ≤50%	Low access—Moderate % healthy
	Low	>50%	Low access—High % healthy
	Moderate	≤25%	Moderate access—Low % healthy
	Moderate	>25% to ≤50%	Moderate access—Moderate % healthy
	Moderate	>50%	Moderate access—High % healthy
	High	≤25%	High access—Low % healthy
	High	>25% to ≤50%	High access—Moderate % healthy
	High	>50%	High access—High % healthy

RHFA represents the percentage (%) of the food environment that is composed of healthy (supermarkets and greengrocers) food outlets within each postal area boundary. * Postal areas with no food retail outlets were classified into one of the RHFA categories using a measure of a larger geographical unit (Statistical Area 2). ** Food environment typology measures drawn from Needham et al.’s [14] cluster analysis of the food environment data; these measures represent potential typology classifications that could be found in the data at the POA level. *** Using a combination of FRAMs overall accessibility was defined as: ‘Low’ if all FRAMs were ‘Low’; ‘Moderate’ where ≥1 FRAMs were ‘Moderate’; and ‘High’ were ≥1 FRAM were ‘High’.

**Table 2:** Characteristics of the Greater Melbourne population sample from the Victorian Population Health Survey (2008, 2011–2012, 2014, and 2017).

		Year			
Characteristics	Categories	2008 (n = 12,526)	2012 (n = 11,246)	2014 (n = 11,760)	2017 (n = 11,713)
**Age (%)**	18–30	12.9	9.0	5.6	15.3
	31–40	17.6	14.2	10.2	15.1
	41–50	19.9	20.3	17.0	15.4
	51–60	18.7	21.2	20.8	17.4
	61–70	16.5	19.4	23.7	19.6
	71+	14.4	16.0	22.8	17.2
**Gender (%)**	Male	39.8	40.5	41.9	47.1
	Female	60.2	59.6	58.1	52.9
**Education (%)**	Primary school/some-high school/other	27.2	21.9	20.7	14.8
	Completed High school/TAFE */trade	37.1	39.3	39.9	36.2
	Tertiary	35.7	38.9	39.4	49.0
**Household income (%)**	<$20,000	13.2	11.1	9.8	4.4
	≥$20 to <40,000	17.1	15.3	17.0	16.4
	≥$40 to <60,000	12.9	12.0	12.0	11.6
	≥$60 to <80,000	11.6	9.7	8.8	9.3
	≥$80 to <100,000	8.1	9.5	8.3	8.6
	≥$100,000+	19.8	25.3	18.3	32.8
	Unknown/not reported	17.4	17.1	25.8	17.0
**Employment status (%)**	Employed **	55.6	56.1	49.2	58.0
	Unemployed	3.1	3.4	3.3	3.7
	Home duties	8.8	6.5	5.0	4.4
	Student	3.3	3.1	2.4	3.8
	Retired	25.7	28.2	37.0	26.8
	Unable to work	3.4	2.6	2.7	2.7
	Other	0.3	0.1	0.5	0.7
**Length of time lived in (%)** neighbourhood/area/council/local government area.	<5 years	27.3	17.4	17.3	32.2
	5–10 years	18.2	17.9	15.9	14.7
	10+ years	54.5	64.8	66.8	53.2
**BMI mean** **(standard deviation)**		26.0 (5.3)	26.5 (5.3)	26.6 (5.2)	26.5 (5.4)

BMI = body mass index. * TAFE: technical and further education—courses in technical and vocational subjects. ** Regarding employment status, ‘employed’ reflects participants that were either employed, self-employed, or reported ‘other-working’.

## Data Availability

The authors were permitted to use the Victorian Population Health Survey data by the Victorian State Government (Australia), which is the custodian of these data. The food environment data used in this study is presented on the Australian Food Retail Environment Monitoring Tool: https://foodenvironmentdashboard.com.au/food-retail/australian-food-retail-environment-monitoring-tool/ (accessed on 16 June 2021).

## Supplementary Materials

The following supporting information can be downloaded at: https://www.mdpi.com/article/10.3390/nu15214503/s1, Table S1: Food outlet descriptions and healthiness scores; Table S2: Proportion of postal areas within each classification of food retail environment measures; Table S3: Summary of food retail environment measures of relative healthy food availability within each food environment typology by postal area in Greater Melbourne between 2008 and 2016; Table S4: Proportion of the Melbourne population sample within each food retail environment measure defined at the postcode level. Table S5: Sidak-adjusted pairwise comparisons of the mean BMI between years and between levels of healthy food outlet accessibility defined at the postcode level in Greater Melbourne, Australia. Table S6: Sidak-adjusted pairwise comparisons of the mean BMI between levels of food environment typology defined at the postcode level in Greater Melbourne, Australia.
