# Supplementary material for: BMI and the Food Retail Environment in Melbourne, Australia: Associations and Temporal Trends
Source: Nutrients. 2023 Oct 24;15(21):4503. doi: 10.3390/nu15214503 (PMC10649206; doi:10.3390/nu15214503)
Supplement: Supplementary file 1 [file nutrients-15-04503-s001.zip › nutrients-2623804-supplementary.pdf]

nutrients

Supplementary Materials

BMI and the Food Retail Environment in Melbourne, Australia: Associations and Temporal Trends

Cindy Needham [1,*], Claudia Strugnell [1,2], Steven Allender [1], Laura Alston [3,4] and Liliana Orellana [5]

Table S1. Food outlet descriptions and healthiness scores.

Food outlet type	Description	Health Score
Fruiterer & greengrocer	Mainly engaged in the sale of fresh fruit and vegetables; including wholesale stores with direct to public sales	10
Fish shop	Mainly engaged in the sale of fresh seafood; including wholesale stores with direct to public sales and takeaway stores also providing a range of fresh seafood.	9
Poultry shop	Mainly engaged in the sale of fresh poultry; including wholesale stores and with direct to public sales.	9
Butchery	Mainly engaged in the sale of fresh meat; including wholesale stores with direct to public sales.	9
Major Supermarket	Mainly engaged in the sale of groceries (fresh foods, canned and packaged foods, dry goods) of non-specialised (conventional) food lines. May contain a butcher or baker. Usually have 5 or more checkouts and a floor area over 1000 square meters. i.e., Woolworths, Coles, BI-LO, Franklins (no frills), ALDI	5
Minor Supermarket	Mainly engaged in the sale of groceries (fresh foods, canned and packaged food, dry goods) of non-specialised (conventional) food lines. Usually have 4 or fewer checkouts and a floor area under 1000 square meters. E.g. Independent grocer or supermarket.	5
Specialty food stores – core foods	Mainly engaged in the sale of a limited line of specialised food such as a particular gourmet food that can be defined under core food.	5
Restaurant/café – franchise	e.g., franchise restaurants and cafes; mainly engaged in the preparation and sale of meals/snacks for consumption on the premises; table service provided; may sell alcohol with food; may provide takeaway services.	0
Restaurant/café – local independent	e.g., restaurant in a golf club, culture-based restaurant/café which is not a take-away such as Mexican, Thai, Chinese etc. ; mainly engaged in the preparation and sale of meals/snacks for consumption on the premises; table service provided; may also sell alcohol with food, may provide takeaway services.	0
Sandwich shop	Mainly engaged in the preparation of filled bread products like sandwiches or rolls.	5
Salad/sushi bar	Mainly engaged in the preparation of salads and sushi.	5
Delicatessen	Mainly engaged in the sale of specialty packaged or fresh products such as cured meats and sausage, pickled vegetables, dips, bread and olives; may also provide dine in meals.	0
Bakery	Mainly oriented towards bread, biscuits, pastries or other flour products with or without packaging.	0
General store	Mainly engaged in the sale of a limited line of groceries generally includes milk, bread and canned and packaged foods.	−5
Specialty food store – extra foods	Mainly engaged in the sale of foods such as ice-creams, donuts, waffles, cakes etc. than can be defined under extra food.	−8
Pub	e.g., pub within a bowing park, pub inside a private gambling club; food primarily engaged in selling alcoholic beverages where consumers can order and consume the alcoholic drinks in premises; can also be part of park or private club.	−5
Take-away local independent	e.g., kebab, fish & chips, burger, chicken shops, local pizza, mainly engaged in the preparation and sale of meals/snacks that are ready for immediate consumption; table service not provided; meals can be eaten on site; taken away or delivered; shop is not a franchise.	−8
Take-away franchise store	e.g., McDonalds, KFC, Subway; mainly engaged in the preparation and sale of meals (excludes donuts, drinks, ice-cream etc.)/snacks that area ready for immediate consumption; table service not provided; meal can be eaten on site, taken away or delivered; the food shop is a franchise/chain store with food being sold in specialised packaging.	−10

Source: Needham et al. 2020 (161): adapted from Moayyed et al. 2017 (68): https://onlinelibrary.wiley.com/doi/abs/10.1111/1747-0080.12286.

Table S2. Proportion of postal areas within each classification of Food retail environment measures.

	Year			
	**2008**	**2012**	**2014**	**2016**
	**N (%)**	**N (%)**	**N (%)**	**N (%)**
**Supermarket accessibility**				
Low	190 (72)	177 (67.1)	176 (66.7)	170 (64.4)
Moderate	26 (9.9)	32 (12.1)	39 (14.8)	40 (15.2)
High	48 (18.2)	55 (20.8)	49 (18.6)	54 (20.5)
**Healthy outlet accessibility**				
Low	154 (58.3)	149 (56.4)	128 (48.5)	144 (54.6)
Moderate	40 (15.2)	39 (14.8)	54 (20.5)	44 (16.7)
High	70 (26.5)	76 (28.8)	82 (31.1)	76 (28.8)
**Unhealthy outlet accessibility**				
Low	90 (34.1)	84 (31.8)	81 (30.7)	82 (31.1)
Moderate	50 (18.9)	48 (18.2)	40 (15.2)	39 (14.8)
High	124 (47)	132 (50)	143 (54.2)	143 (54.2)
**Food Environment Typology**				
Low access – Low % healthy	86 (32.58)	69 (26.1)	57 (21.6)	67 (25.4)
Low access – Moderate % healthy	16 (6.06)	22 (8.3)	28 (10.6)	23 (8.7)
Low access – High % healthy	7 (2.65)	7 (2.7)	10 (3.8)	6 (2.3)
Moderate access – Low % healthy	25 (9.47)	31 (11.7)	23 (8.7)	24 (9.1)
Moderate access – Moderate % healthy	9 (3.41)	7 (2.7)	12 (4.6)	17 (6.4)
Moderate access – High % healthy	0 (0.0)	0 (0.0)	0 (0.0)	0 (0.0)
High access – Low % healthy	44 (16.67)	44 (16.7)	43 (16.3)	47 (17.8)
High access – Moderate % healthy	77 (29.17)	84 (31.8)	91 (34.5)	80 (30.3)
High access – High % healthy	0 (0.0)	0 (0.0)	0 (0.0)	0 (0.0)

Healthy, Less Healthy, Unhealthy: Low Access < 1 outlet per km^2, Moderate Access ≥ 1 outlet per km^2; High Access ≥ 2 outlet per km^2. Supermarkets: Low Access = < 0.625 outlet per km^2, Moderate Access ≥ 0.625 outlets per km^2; High Access ≥ 1.25 outlet per km^2.RHFA: represents the percentage (%) of the food environment that is composed of healthy (supermarkets and green grocers) food outlets within each postal area boundary. Total number of Postal Areas within each study years = 264.

Table S3. Summary of Food Retail Environment measures of Relative Healthy Food Availability within each Food environment typology by Postal Area in Greater Melbourne between 2008 and 2016.

	Mean (SD)											
	**2008**	**2012**	**2014**	**2016**	**2008**	**2012**	**2014**	**2016**	**2008**	**2012**	**2014**	**2016**
	**Low access - Low % healthy**				**Moderate access - Low % healthy**				**High access - Low % healthy**			
Supermarkets per km2	0.07(0.1)	0.08(0.11)	0.08(0.13)	0.07(0.11)	0.15(0.13)	0.27(0.19)	0.26(0.2)	0.34(0.21)	0.44(0.45)	0.69(0.59)		
Healthy per km2	0.18(0.22)	0.17(0.22)	0.21(0.27)	0.14(0.20)	0.54(0.35)	0.59(0.36)	0.72(0.37)	0.68(0.31)	1.58(1.38)	1.74(1.31)	2.16(2.13)	1.87(1.66)
Less Healthy per km2	0.64(1.69)	0.58(1.8)	0.38(0.34)	0.41(0.47)	1.73(1.36)	1.75(1.20)	2.33(3.43)	1.53(0.56)	10.65(15.81)	11.12(15.07)	11.8(17.02)	12.08(16.68)
Unhealthy per km2	0.63(0.63)	0.56(0.53)	0.61(0.63)	0.65(1.12)	1.8(0.55)	1.76(0.70)	1.88(0.80)	2.19(0.67)	5.25(4.98)	5.82(5.35)	6.23(6.40)	6.44(6.25)
RHFA (% healthy)	10.05(9.45)	10.89(10.52)	9.19(11.23)	8.49(9.9)	14.84(9.36)	18.62(9.21)	17.9(10.57)	17.27(7.53)	16.72(7.06)	18.51(6.49)	17.75(5.76)	17.86(5.80)
	**Low access - Moderate % healthy**				**Moderate access - Moderate % healthy**				**High access - Moderate % healthy**			
Supermarkets per km2	0.12(0.15)	0.15(0.16)	0.13(0.10)	0.15(0.10)	0.34(0.18)	0.36(0.22)	0.4(0.17)	0.48(0.25)	0.82(0.38)	1.03(0.65)	0.97(0.57)	1.09(0.66)
Healthy per km2	0.24(0.21)	0.29(0.24)	0.3(0.23)	0.27(0.17)	1.14(0.18)	0.87(0.28)	0.95(0.33)	0.95(0.36)	2.6(1.41)	2.61(1.95)	2.83(1.67)	2.8(1.57)
Less Healthy per km2	0.5(0.41)	0.57(0.48)	0.47(0.38)	0.52(0.41)	1.59(0.26)	1.53(0.44)	1.8(1.03)	2.29(3.50)	7.03(5.74)	7.06(7.37)	6.92(6.24)	7.64(7.18)
Unhealthy per km2	0.35(0.28)	0.43(0.34)	0.49(0.34)	0.48(0.31)	1.54(0.32)	1.42(0.30)	1.44(0.51)	1.6(0.65)	3.66(2.29)	4.01(2.97)	4.1(2.41)	4.26(2.65)
RHFA (% healthy)	39.31(9.21)	36.31(7.75)	34.38(6.46)	34.48(7.58)	36.35(9.58)	31.77(6.58)	36.06(8.24)	34.25(7.58)	35.09(7.00)	34.02(6.39)	34.21(7.20)	34.17(7.79)
	**Low access - High % healthy**											
Supermarkets per km2	0.13(0.17)	0.08(0.09)	0.05(0.06)	0.07(0.09)								
Healthy per km2	0.19(0.25)	0.11(0.1)	0.06(0.06)	0.09(0.09)								
Less Healthy per km2	0.53(1.05)	0.25(0.26)	0.1(0.20)	0.22(0.45)								
Unhealthy per km2	0.06(0.11)	0.05(0.10)	0.02(0.02)	0.02(0.02)								
RHFA (% healthy)	85.71(17.82)	84.35(19.77)	82.55(18.97)	83.33(18.26)								

Healthy, Less Healthy, Unhealthy: Low Access < 1 outlet per km^2, Moderate Access ≥ 1 outlet per km^2; High Access ≥ 2 outlet per km^2. Supermarkets: Low Access = < 0.625 outlet per km^2, Moderate Access ≥ 0.625 outlets per km^2; High Access ≥1.25 outlet per km^2.RHFA: represents the percentage (%) of the food environment that is composed of healthy (supermarkets and green grocers) food outlets within each postal area boundary. Total number of Postal Areas within each study years = 264.

Table S4. Proportion of the Melbourne population sample within each food retail environment measure defined at the postcode level.

	Year			
	**2008 (N = 12,526) (%)**	**2012 (N = 11,246) (%)**	**2014 (N = 11,760) (%)**	**2016 (N = 11,713) (%)**
**Relative Healthy Food Availability**				
zero	3.1	3.6	2.2	2.2
≤ 25%	64.2	57.9	44.1	55.3
> 25 & ≤ 50%	31.0	37.3	52.0	40.9
> 50%	1.7	1.3	1.8	1.6
**Supermarket accessibility**				
Low	78.1	71.3	71.1	66.9
Moderate	13.1	16.2	17.6	18.8
High	8.8	12.6	11.3	14.3
**Healthy outlet accessibility**				
Low	60.2	56.9	48.0	54.3
Moderate	20.2	20.1	25.4	21.0
High	19.6	23.0	26.6	24.8
**Less Healthy outlet accessibility**				
Low	35.2	29.5	30.4	27.6
Moderate	18.5	19.5	18.2	20.5
High	46.4	51.0	51.4	51.9
**Unhealthy outlet accessibility**				
Low	28.3	26.3	24.6	24.4
Moderate	24.7	21.2	17.9	17.0
High	47.1	52.5	57.5	58.6
**Food Environment Typology**				
Low access - High % healthy	1.1	0.9	1.7	1
Low access - Moderate % Healthy	3.3	9	14.4	8.9
Low access - Low % healthy	33.1	22.7	15.4	20.8
Moderate access - Moderate % healthy	4.9	3.2	5.2	7
Moderate access - Low % healthy	12.3	15.5	9.9	11.7

High access - Moderate % healthy	24.3	25.4	31.3	26.2
High access - Low % healthy	21	23.4	22.2	24.5

Healthy, Less Healthy, Unhealthy: Low Access < 1 outlet per km^2, Moderate Access ≥ 1 outlet per km^2; High Access ≥ 2 outlet per km^2. Supermarkets: Low Access = < 0.625 outlet per km^2, Moderate Access ≥ 0.625 outlets per km^2; High Access ≥ 1.25 outlet per km^2.

Relative Healthy Food Availability: represents the proportion (%) of the food environment that is composed of healthy (supermarkets and green grocers) food outlets within each postal area boundary.

Table S5. Sidak adjusted pairwise comparisons of the mean BMI between years and between levels of Healthy food outlet accessibility defined at the postcode level in Greater Melbourne, Australia.

**Healthy**	**Healthy**	**Supermarkets**	**Unhealthy**
Between food environment measure levels		**Contrast (95%CI)**	
High vs Low	**−0.68(−0.94, −0.43)**	**−0.32(−0.56, −0.07)**	0.16(−0.11, 0.44)
Moderate vs Low	**−0.34(−0.57, −0.12)**	**−0.33(−0.63, −0.04)**	**−0.38(−0.64, −0.12)**
High vs Moderate	**−0.34(−0.6, −0.08)**	−0.02(−0.33, 0.29)	**−0.54(−0.78, −0.3)**
Between levels year			
2012 vs 2008	**0.37(0.18, 0.57)**	**0.35(0.1, 0.6)**	**0.44(0.26, 0.63)**
2014 vs 2008	**0.37(0.18, 0.57)**	**0.31(0.06, 0.57)**	**0.46(0.27, 0.66)**
2016 vs 2008	**0.56(0.36, 0.76)**	**0.52(0.27, 0.77)**	**0.71(0.51, 0.9)**
2014 vs 2012	0(−0.2, 0.19)	−0.04(−0.28, 0.2)	0.02(−0.18, 0.22)
2016 vs 2012	0.19(−0.01, 0.39)	0.17(−0.06, 0.4)	**0.26(0.06, 0.47)**
2016 vs 2014	0.19(0, 0.38)	0.21(−0.03, 0.44)	**0.25(0.04, 0.45)**

Model: BMI Mean estimates and 95% confidence intervals obtained under linear mixed models including postal area as a random effect; and the Food environment measure (Healthy, Unhealthy food outlets, Supermarkets) interaction x year, adjusted by Age, Gender, Education, Employment Status, Household Income and Length of time lived in local area.

Pairwise comparisons presented only for measures for which the interaction measure x year was not significant. Bold: $p \leq 0.05$.

Measure of accessibility for Healthy: Low Access < 1 outlet per km^2, Moderate Access ≥1 outlet per km^2; High Access ≥2 outlet per km^2.

Table S6. Sidak adjusted pairwise comparisons of the mean BMI between levels of food environment typology defined at the postcode level in Greater Melbourne, Australia.

Food Environment Typology	Contrast [95% CI]
Low access - Low % healthy vs Low access - High % healthy	0.18(−0.65, 1.02)
Low access - Moderate % healthy vs Low access - High % healthy	0.08(−0.78, 0.95)
Low access - Moderate % healthy vs Low access - Low % healthy	−0.10(−0.49, 0.29)
Low access - Moderate % healthy vs Moderate access - Low % healthy	0.06(−0.42, 0.55)
Low access - Moderate % healthy vs Moderate access - Moderate % healthy	0.33(−0.23, 0.90)
Low access - Moderate % healthy vs High access - Low % healthy	**0.73(0.27, 1.20)**
Low access - Moderate % healthy vs High access - Moderate % healthy	**0.63(0.19, 1.08)**
Moderate access - Low % healthy vs Low access - High % healthy	0.02(−0.84, 0.88)
Moderate access - Low % healthy vs Low access - Low % healthy	−0.16(−0.55, 0.22)
Moderate access - Moderate % healthy vs Low access - High % healthy	−0.25(−1.16, 0.66)
Moderate access - Moderate % healthy vs Moderate access - Low % healthy	−0.27(-0.77, 0.23)
Moderate access - Moderate % healthy vs High access - Low % healthy	0.40(−0.08, 0.89)
Moderate access - Moderate % healthy vs High access - Moderate % healthy	0.30(−0.17, 0.77)
Moderate access - Moderate % healthy vs Low access - Low % healthy	−0.43(−0.93, 0.06)
High access - Low % healthy Vs Low access - High % healthy	−0.65(−1.49, 0.18)
High access - Low % healthy vs Low access - Low % healthy	**−0.84(−1.20, −0.47)**
High access - Low % healthy vs Moderate access - Low % healthy	**−0.67(−1.05, −0.29)**
High access - Moderate % healthy vs Low access - High % healthy	−0.55(−1.37, 0.28)
High access - Moderate % healthy vs Low access - Low % healthy	**−0.73(−1.08, −0.39)**
High access - Moderate % healthy vs Moderate access - Low % healthy	**−0.57(−0.94, −0.20)**
High access - Moderate % healthy vs High access - Low % healthy	0.10(−0.18, 0.39)

Model: BMI Mean estimates and 95% confidence intervals obtained under linear mixed models including postal area as a random effect; and the Food environment measure (Healthy, Unhealthy food outlets, Supermarkets) interaction x year, adjusted by Age, Gender, Education, Employment Status, Household Income and Length of time lived in local area.

Bold: $p \leq 0.01$.

Food environment typology reflects postcodes grouped by similarities across Relative Healthy Food Access; and, Food Retail Accessibility Measures related to Supermarkets, Healthy, Less Healthy and Unhealthy food outlets per km^2.
